# Electrical and optical enhancement of ITO/Mo bilayer thin films via laser annealing

**DOI:** 10.3762/bjnano.13.133

**Published:** 2022-12-28

**Authors:** Abdelbaki Hacini, Ahmad Hadi Ali, Nurul Nadia Adnan, Nafarizal Nayan

**Affiliations:** 1 Laser and Semiconductor Technology Research Group, COR PDSR, Department of Physics and Chemistry, Faculty of Applied Sciences and Technology, Pagoh Educational Hub, Universiti Tun Hussein Onn Malaysia, 84600 Pagoh, Johor, Malaysia; 2 Microelectronic and Nanotechnology Shamsuddin Research Centre (MiNT-SRC), Universiti Tun Hussein Onn Malaysia, 86400 Parit Raja, Batu Pahat, Johor, Malaysia

**Keywords:** indium tin oxide (ITO), laser annealing, molybdenum (Mo), Nd:YAG

## Abstract

ITO/Mo bilayer thin films were sputtered on n-type silicon and glass substrates and annealed with a Nd:YAG pulsed laser. The structural results show that both the as-deposited and the annealed ITO/Mo thin films have a polycrystalline structure, and that the annealing treatment enhanced the crystallinity of samples. Moreover, the XRD patterns exhibited a cubic structure preferentially oriented along the (222) and (400) planes. The AFM analysis shows that grain size and RMS roughness increased from 16.02 to 36.19 nm and 0.4 to 2.6 nm, respectively, when the laser energy was increased to 120 mJ. The as-deposited sample has an optical transmittance of nearly 80% in the 300–800 nm range. The laser annealing yielded a higher transmittance of 94% and increased the bandgap energy from 2.76 to 2.88 eV at 120 mJ. The annealing treatment decreased the resistivity from 15.63 × 10^−4^ to 1.73 × 10^−4^ Ω/cm^−1^. Additionally, the figure of merit of the ITO/Mo structure improved significantly from 6.63 × 10^−4^ Ω^−1^ of the as-deposited sample to 17.6 × 10^−3^ Ω^−1^ of the the annealed structure. The results indicate that the laser annealing could improve the efficiency of the transparent conductive layer, which can be potentially applied in optoelectronic devices.

## Introduction

Transparent conducting (TC) materials have been used in many optoelectronic devices, including flat panel displays [1], light-emitting diodes [2], heat-reflecting mirrors [3], anti-reflective coatings [4], gas sensors [5], and solar cells [6]. For this purpose, many materials have been developed to improve the performance of optoelectronic devices. One of these materials is indium tin oxide (ITO), which combines high transparency with high conductivity [7–8]. However, a single layer of as-deposited ITO shows a high resistivity. Consequently, inserting a very thin metal film layer can significantly improve the electrical properties without affecting the optical transmission [9]. Several metals have been used for such thin layers, including silver [10], aluminium [11], copper [12], and gold [13]. Molybdenum thin films are another choice for the application in solar cells because of good adherence to the substrate, very high thermal stability (up to 600 °C), and high electrical conductivity [14].

Over the last decades, the development of solar cells has grown dramatically. The cells have become larger, thinner, and lighter. This increases the electrical resistivity, which is undesirable. The concentration and mobility of charge carriers need to be increased to overcome this problem. Furthermore, enhancing electrical and optical performance at the same time is a critical research topic that has many challenges. Generally, the physical properties of TC materials are essentially affected by many factors, such as the type of substrate [15], the deposition technique [16–18], the deposition conditions [19–22], and the annealing treatment [23]. Among these factors, heat treatment is a significant factor in rearranging the nanostructure, removing defects, and improving the thin film’s quality [9]. Conventional annealing treatment requires long periods of time and high temperatures during operation [24]. This led to the search for new methods using lower temperatures and shorter process times. Laser annealing can fulfil these requirements [24–26]. During laser annealing, a laser beam irradiates the surface layer of the thin film without causing damage. The temperature increases quickly during the process within a short period [24,27]. The laser beam must have a uniform intensity with asymmetrical distribution to obtain surfaces of good morphology with low roughness.

In this work, we investigate the effect of laser annealing treatment on ITO/Mo (IM) bilayer thin films as transparent conducting material for solar cell applications. The structural, optical, and electrical properties of the IM structure were examined as functions of the laser energy.

## Experimental

IM bilayer thin films were sputtered on n-type silicon(111) substrates for structural, morphological, and electrical characterization. In addition, the bilayer thin films were deposited on glass substrates for optical characterization. This process was performed using RF magnetron sputtering (Quorum Q300T D) at 10^−4^ mbar pressure. The magnetron sputter contains a double target with high purity (approx. 99.99%). The first target is for sputtering ITO (90 wt % In_2_O_3_ and 10 wt % SnO_2_), and the second target is for sputtering Mo. Before deposition, the Si and the glass samples were cut into pieces and cleaned to suit the characterization equipment. The Si substrates were ultrasonically cleaned using acetone, isopropyl alcohol, and distilled water. The glass substrates were cleaned using Decon-90 cleaner. The glass and Si samples were dried with nitrogen gas and treated for 10 min in a plasma cleaner. In the IM structure, the thickness of ITO was 125 nm while the thickness of Mo was 10 nm. The thickness was controlled by two quartz crystal balances integrated within the chamber. After deposition, the bilayer thin film was treated using a Nd:YAG pulsed laser with a wavelength of 1064 nm and a repetition rate of 1 Hz. Different energies from 80 to 240 mJ with a step of 40 mJ were used. The samples were placed in a closed metal cube under nitrogen during annealing to avoid contamination and interaction with particles. A converging lens was placed between the laser and the sample, and the samples were placed behind the focal plane of the lens (low intensity and big spot).

The crystalline properties of the films were determined using X-ray diffraction (PANalytical diffractometer, λ = 1.5406 Å). The XRD measurements were carried out in 2θ mode between 20° and 80°. Topology and roughness of the bilayer structure were studied utilizing an atomic force microscope (AFM, Bruker Dimension Edge) and the Gwyddion software. The optical transmission was measured using an UV–vis spectrophotometer (UV-3600i Plus, SHIMADZU) in the range of λ = 300–800 nm. Finally, the electrical properties were determined using a four-point probe system.

## Results and Discussion

**Figure 1:**
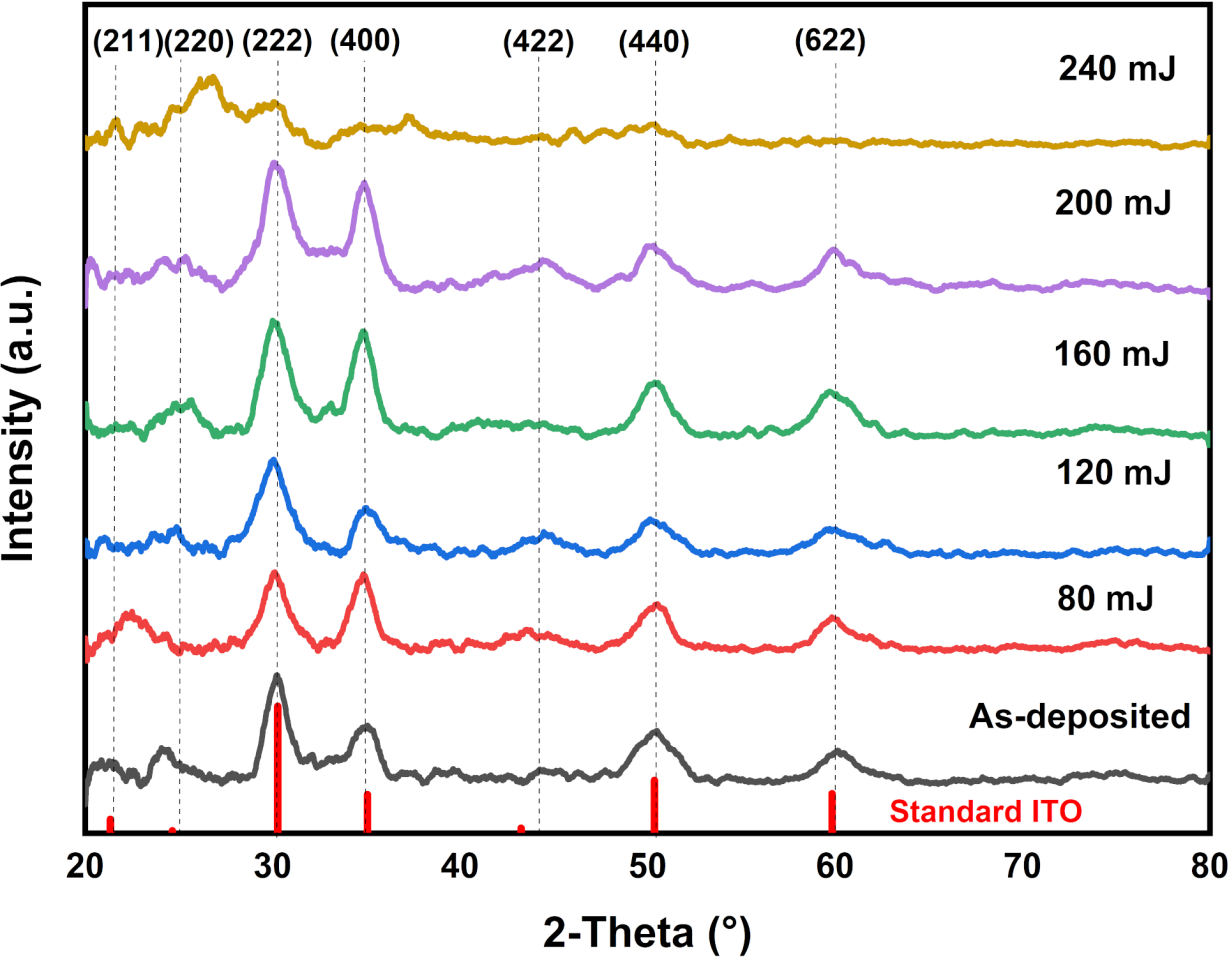
XRD patterns of ITO/Mo bilayer thin films sputtered on n-type silicon after laser annealing with different energies: as-deposited, 80, 120, 160, 200, and 240 mJ.

The crystalline structure of the ITO/Mo bilayer thin film after laser annealing was investigated using XRD. Figure 1 shows the structural evolution for the as-deposited and the samples annealed with different laser energies. Most samples show a polycrystalline structure crystallized in a cubic structure (JCPDS:71-2194) [28]. However, the sample treated with 240 mJ shows an amorphous structure because of the high energy that diffuses gas atoms from the chamber into the surface [29]. The obtained peaks correspond to ITO. It can be seen that the predominant peaks are (222) and (400), which are the preferred orientations before and after laser annealing. The intensities of the crystallite peaks orientation increased and attained a maximum at 200 mJ.

The crystallite size (*D*) and the dislocation density (δ) were determined from the XRD peaks using the following equations [28]:


[1] $$D=\frac{0.9\lambda }{\beta \cos\theta_{\beta }},$$

[2] $$\delta =\frac{1}{D^{2}},$$

where *D*, 0.9, λ, β, and δ are the crystallite size, the shape factor, the wavelength of the incident X-rays, the full width at half maximum of the reflection at the diffraction angle θ_β_, and the dislocation density, respectively. The average crystallite size increased with the laser energy up to 200 mJ. It decreased to reach a minimum at an energy of 240 mJ. This trend in crystallite size is due to the rearrangement of the nanostructure. The particles sizes increase, defects are reduced, and the dislocation density (δ) decreases from 6.53 × 10^14^ to 3.21 × 10^14^ lines/m^2^.

**Figure 2:**
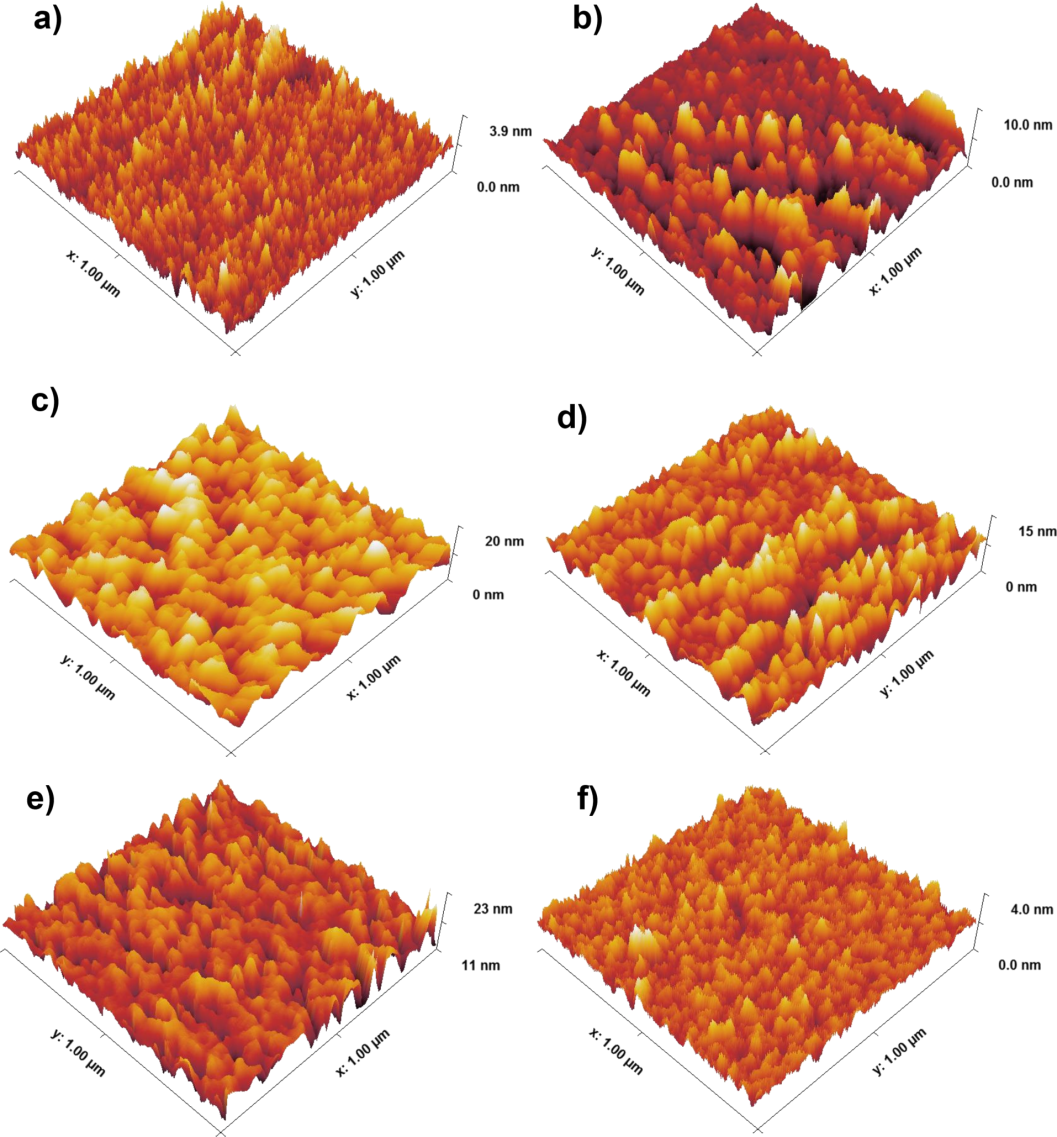
AFM images of ITO/Mo bilayer thin films. (a) As deposited and treated with (b) 80, (c) 120, (d) 160, (e) 200, and (f) 240 mJ.

**Figure 3:**
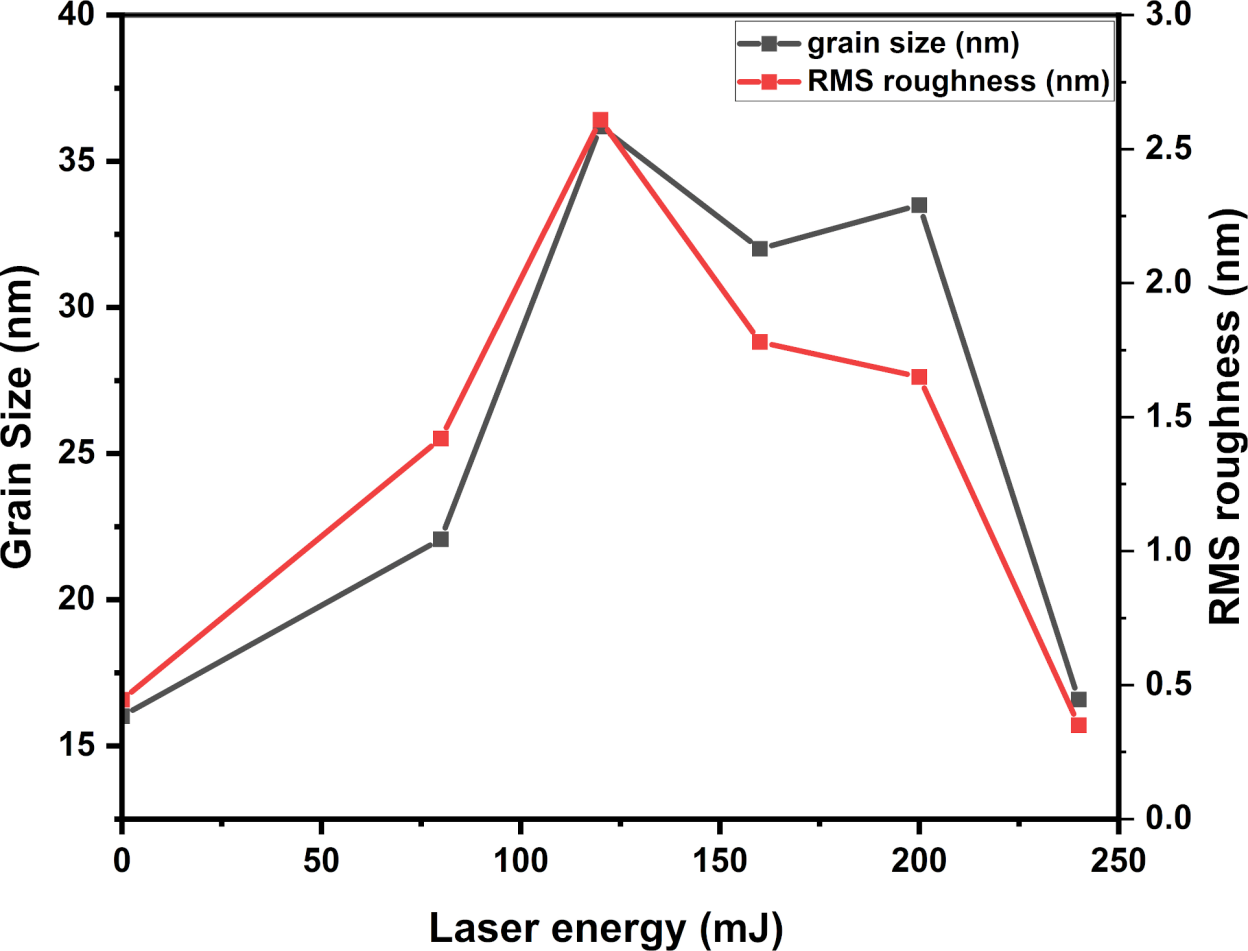
The mean roughness RMS and the grain size of the ITO/Mo bilayer thin films annealed using different laser energies.

Figure 2 displays the surface morphology in 3D images of ITO/Mo for the as-deposited and annealed samples. These images were scanned over an area of 1.0 × 1.0 µm using AFM. Figure 2 shows the different morphologies for as-deposited and annealed samples using a laser for different energies. It can be observed that size and distribution of the grains change with laser annealing energy. Figure 3 exhibits the grain size and RMS roughness as functions of the laser annealing energy. As the laser energy increased from 0 to 120 mJ, the grain size increased from 16.1 to 36.2 nm. This increase is due to the effect of the laser on the surface reducing defects and eliminating grain boundaries. Above 120 mJ, the grain size decreased with the increase of the laser energy to 16.6 nm for 240 mJ. This decrease is due to the diffusion of gas atoms from the chamber into the upper layer. Simultaneously, the RMS roughness increased from 0.44 to 2.61 nm for the as-deposited sample and the one treated with 120 mJ, respectively. After that, the RMS roughness decreased to 0.35 nm for the sample treated with 240 mJ.

**Figure 4:**
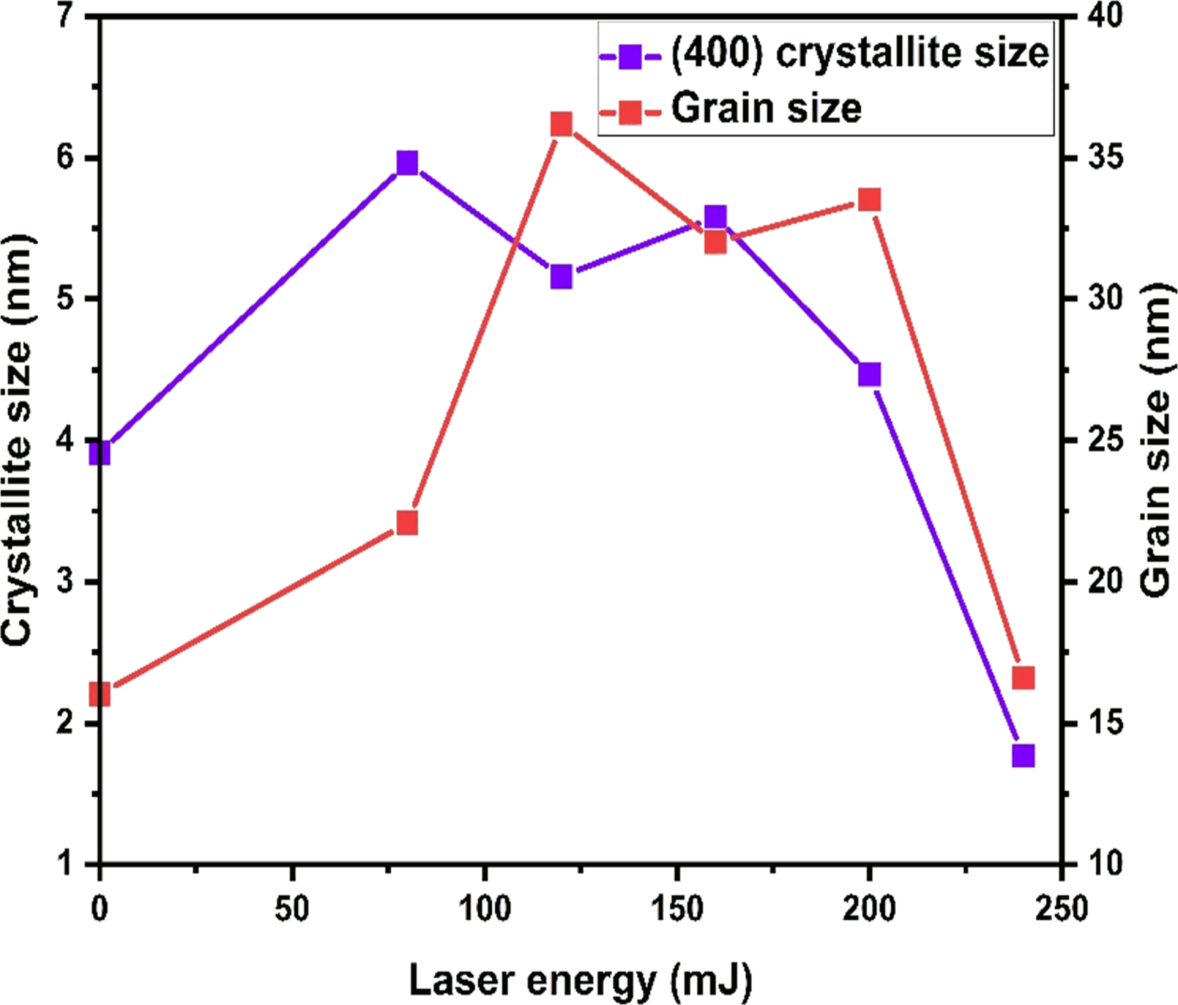
The grain size and the crystallite size in the (400) orientation extracted from XRD results of the ITO/Mo bilayer thin films treated with different laser energy.

The changes in size and distribution of grains agree with the variation of crystallite size obtained by XRD analysis. Figure 4 shows the correlation between the grain size evolution and crystallite size corresponding to the (400) orientation. This variation is due to the rearrangement of the crystallites during the laser annealing of the IM bilayer thin film structure. Moreover, both analyses show that the ITO/Mo has a polycrystalline structure. As well, it can be seen from the grain size analysis that the energy of 120 mJ gives the highest grain size and good crystallinity because most of the orientation of the crystallites appears in the XRD results.

**Figure 5:**
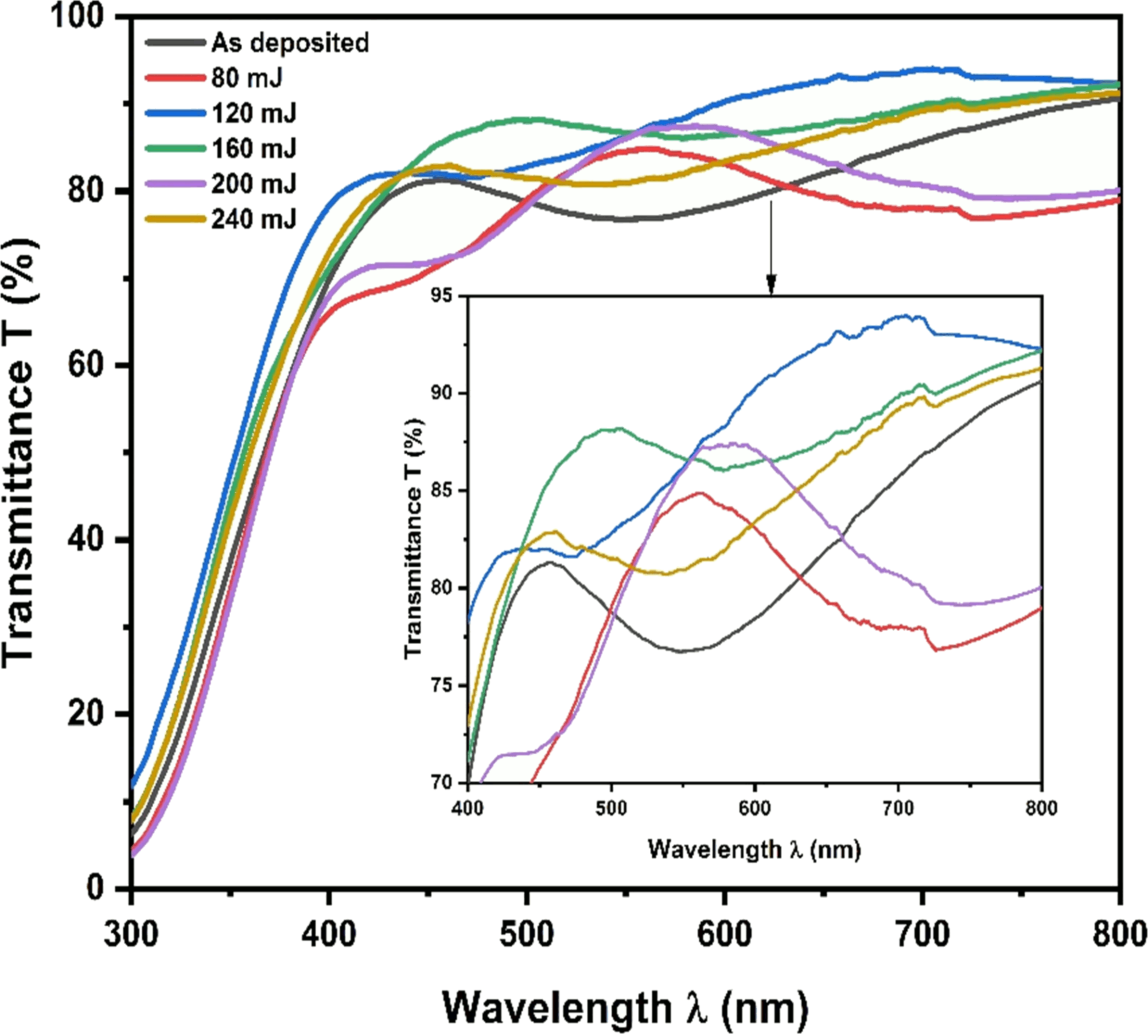
The transmittance of annealed ITO/Mo bilayer thin film.

Figure 5 shows the optical transmittance spectra of the as-deposited and annealed ITO/Mo bilayer structure thin films. The measurement was taken in the wavelength range of 300–800 nm. The as-deposited sample shows the lowest optical transmittance with an average close to 80% at a wavelength of 550 nm. The annealed samples exhibit an increase in the transmittance with the increase of laser energy until they attain a maximum of 94% for 120 mJ. Above 120 mJ, the optical transmission decreases with the increase of the energy up to 240 mJ. This increase in transmittance is due to the improvement in crystallinity caused by the laser. The crystalline improvement leads to less light scattering in the metal layer [29–30]. Moreover, laser annealing reduces the defects, including grain boundaries and impurities, reducing light scattering and photon–electron interactions [29–31].

The optical bandgap energy *E*_g_ of ITO/Mo thin film was studied before and after laser annealing. The bandgap energy *E*_g_ was determined using the following equation (Tauc relation) [28]:


[3] $$\alpha h\nu =A{\left(h\nu -E_{\text{g}}\right)}^{n},$$

where α is the absorption coefficient, *h*ν is the photon energy; *A* is a constant, *E*_g_ is the bandgap energy, *n* = 0.5 for a direct bandgap, and *n* = 2 for an indirect bandgap. The bandgap energy values of the ITO/Mo thin films were calculated and listed in Table 1. The bandgap energy increased from 2.76 to 2.88 eV, corresponding to 0–120 mJ laser energy. Then it decreased to 2.79 for 240 mJ. This result clearly shows that laser annealing affects significantly the transmittance and the optical bandgap.

**Table 1:** Bandgap values of the laser-annealed ITO/Mo bilayer thin films.

Laser energy (mJ)	0	80	120	160	200	240

Bandgap energy (eV)	2.76	2.87	2.88	2.88	2.81	2.79

**Figure 6:**
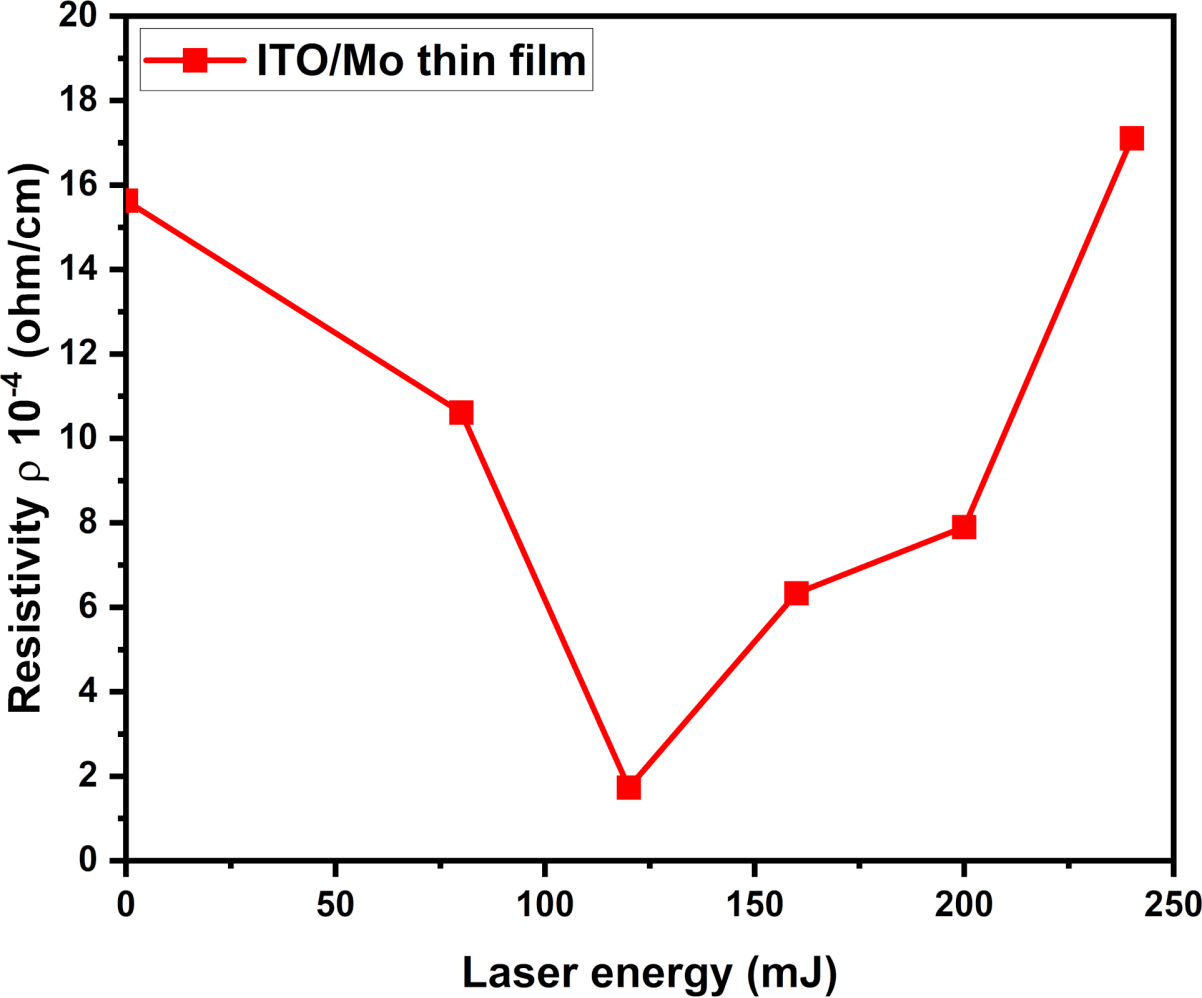
ITO/Mo bilayer resistivity measured of thin films deposited on silicon as a function of the laser annealing energy.

Electrical properties of ITO/Mo and ITO films obtained from four-point probe measurements are shown in Figure 6. The results exhibit a decrease in the resistivity from 15.63 × 10^−4^ to 1.73 × 10^−4^ Ω/cm, for the as-deposited sample and the sample annealed at 120 mJ. An increase in resistivity follows for higher annealing energies. The resistivity variation can be explained by the behavior of the metal–semiconductor contact and the effect of laser annealing on the structural defects of the surface. The inclusion of a thin metal film with low resistivity and the reduction of grain boundaries lead to a decrease in resistivity. Moreover, the annealing treatment minimizes the trade-off between transparency and conductivity [9].

**Figure 7:**
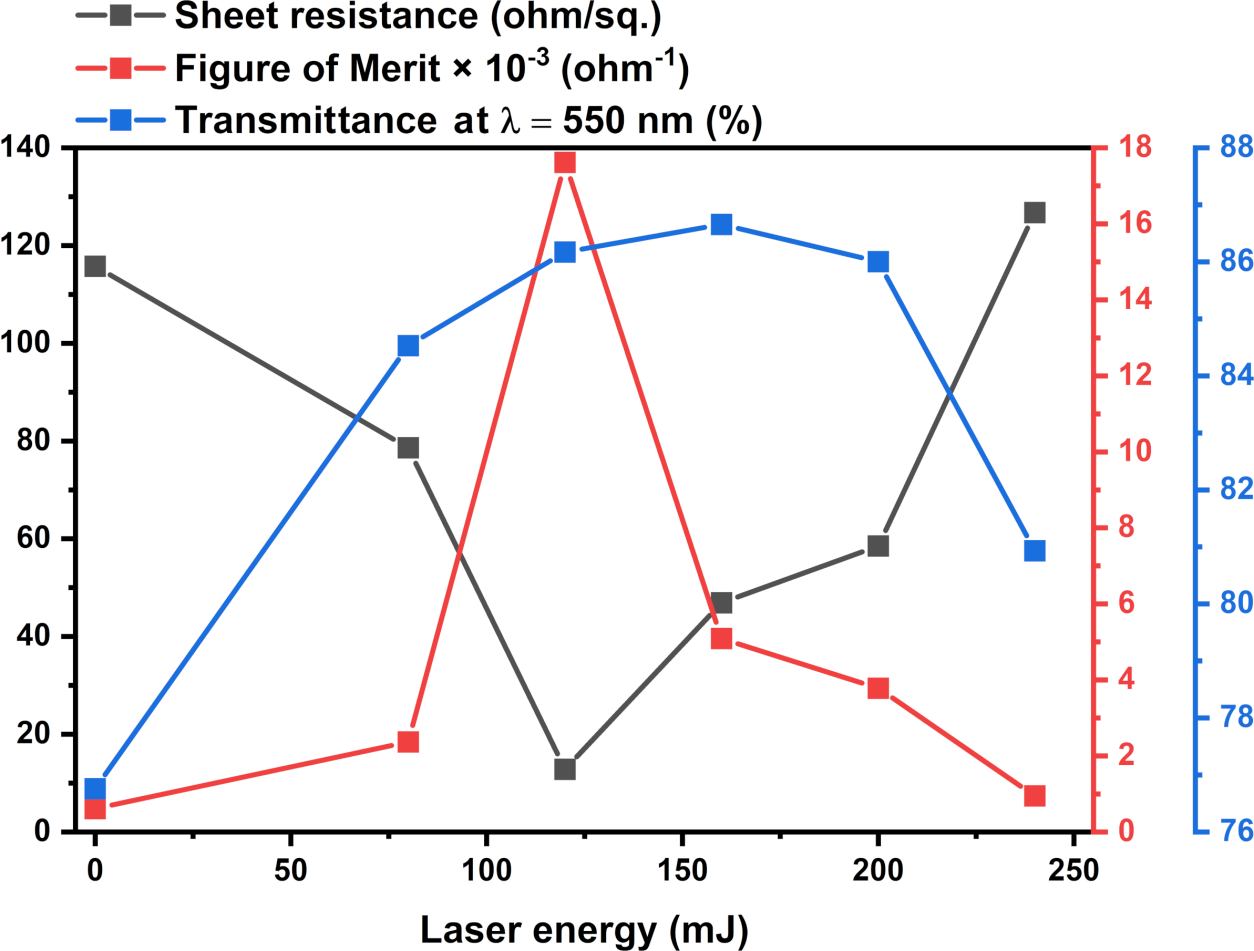
*R*_s_ sheet resistance, *T* transmittance at 550 nm, and FOM of ITO/Mo bilayer films as functions of the laser annealing energy.

The sheet resistance of the ITO/Mo films can be expressed as [29]:


[4] $$R_{\text{s}}=\frac{\rho }{t},$$

where *R*_s_ is the sheet resistance, ρ is the resistivity, and *t* is the thickness (=135 nm). The lowest sheet resistance value obtained is 128.14 Ω/sq from the sample annealed with 120 mJ, as shown in Figure 7. The sheet resistance has the same trend as the resistivity. Moreover, the optoelectronic properties of the IM films are better compared to the ones fabricated in the work of Ali et al. [32] who used ITO/Ag and ITO/Ni bilayers.

The figure of merit (FOM) is a quantitative value that can evaluate the performance of the device. From the sheet resistance (*R*_s_) and the optical transmittance (*T*), the quality of the thin film can be calculated using the FOM relation in Equation 5 [33–35]:


[5] $$\text{FOM}=\frac{T^{10}}{R_{\text{s}}}.$$

It can be noted that the figure of merit increased with the increase of laser energy from 0 to 120 mJ. This increase is due to the simultaneous improvement of transmittance and resistivity. The highest value obtained is 17.6 × 10^−3^ Ω^−1^ after annealing at 120 mJ. Above 120 mJ, the figure of merit decreased with the increase of laser energy. Consequently, the optimal laser energy can be estimated as 120 mJ for the ITO/Mo bilayer structure from the presented electrical and optical results.

## Conclusion

ITO/Mo bilayer thin film structures were deposited on glass and silicon substrates using RF magnetron sputtering and, subsequently, investigated. The films were annealed using a Nd:YAG pulsed laser with different energies. The inclusion of thin Mo films and annealing at 120 mJ improved the structural, morphological, optical, and electrical properties. The XRD results show a good crystallinity for the annealed IM bilayer thin film in a polycrystalline cubic structure oriented along the (222) and (400) planes. The analysis of AFM shows a significant increase in grain size after laser annealing from 16.02 to 36.19 nm. The as-deposited ITO/Mo shows a high transmittance of 81.32%. After the annealing treatment, the optical transmittance increases significantly to 94.04% for the 120 mJ of laser energy. The bandgap energy was increased from 2.76 to 2.88 eV. Additionally, the annealing treatment yields an essential improvement in the resistivity, which decreased from 15.63 × 10^−4^ to 1.73 × 10^−4^ Ω/cm^−1^. The figure of merit of the ITO/Mo structure showed a significant enhancement from 6.63 × 10^−4^ to 17.6 × 10^−3^ Ω^−1^. The results prove that laser annealing enhances the optoelectronic efficiency properties of the thin film. This can be applied to many applications, including solar cells, flat panels, LEDs, and gas sensors.
